# Supplementary material for: Linear ubiquitination regulates the KSHV replication and transcription activator protein to control infection
Source: Nat Commun. 2024 Jun 29;15:5515. doi: 10.1038/s41467-024-49887-6 (PMC11217414; doi:10.1038/s41467-024-49887-6)
Supplement: Supplementary file 1 — Supplementary Information [file 41467_2024_49887_MOESM1_ESM.pdf]

SUPPLEMENTARY MATERIALS

Linear ubiquitination regulates the KSHV replication and transcription activator protein to control infection

Yi Luan[1,2,3,4#], Wenying Long[5#], Lisi Dai[6,7,8], Panfeng Tao[9], Zhifen Deng[1,2,3,4], Zongping Xia[1,2,3,4]*

[1]Clinical Systems Biology Laboratories, Translational Medicine Center, The First Affiliated Hospital of Zhengzhou University, Zhengzhou, 450052, Henan, China.

[2]Institute of Infection and Immunity, Henan Academy of Innovations in Medical Science, Zhengzhou, China.

[3]Department of Neurology, the First Affiliated Hospital of Zhengzhou University, Zhengzhou, Henan, China.

[4]NHC Key Laboratory of Prevention and Treatment of Cerebrovascular Diseases, the First Affiliated Hospital of Zhengzhou University, Zhengzhou, Henan, China.

[5]Center for Clinical Research, the Fourth Affiliated Hospital of School of Medicine, and International School of Medicine, International Institutes of Medicine, Zhejiang University, Yiwu, 322000, Zhejiang, China.

[6]Department of Pathology & Pathophysiology of Second Affiliated Hospital, Zhejiang University School of Medicine, Hangzhou, Zhejiang, China.

[7]Department of Surgical Oncology of Second Affiliated Hospital, Zhejiang University School of Medicine, Hangzhou, Zhejiang, China.

[8]School of Basic Medical Sciences, Zhejiang University, China.

[9]Life Sciences Institute, Zhejiang University, Hangzhou 310058, Zhejiang, China.

[#] These authors have contributed equally to this work.

*Correspondence to: Zongping Xia (email: zxia2018@zzu.edu.cn).

Short title: RTA linear ubiquitination and KSHV infection

Keywords: RTA, KSHV, linear ubiquitination, LUBAC, OTULIN.

Supplementary Figures

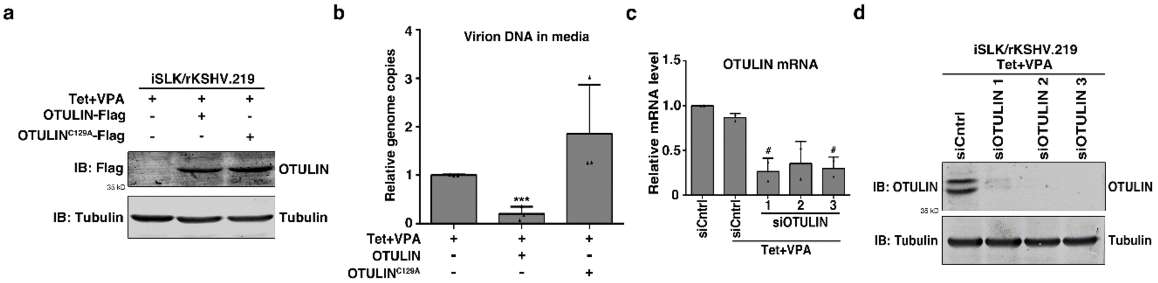

Supplementary Fig. 1. OTULIN regulates KSHV lytic reactivation (Related to Fig. 1). (**a** and **b**) iSLK/rKSHV.219 cells were transfected with vector, OTULIN, or OTULIN^{C129A} for one day, followed by treatment with Tet plus VPA for two days. Protein expression levels of transfected OTULIN were detected by western blotting (a). Culture media were collected for DNA extraction and used for viral DNA quantification using qPCR (b), **, *P* < 0.01 by two-sided *t*-test versus the Tet+VPA group, n = 3 biological replicates and 2 technical replicates, mean ± s.d., two-sided t test, * *P* = 0.00035 (Tet/VPA vs. Tet/VPA+OTULIN). (**c** and **d**) iSLK/rKSHV.219 cells were transfected with control siRNA (siCntrl) or three different pairs of siRNAs targeting OTULIN (siOTULIN 1, 2 and 3). After three days, the cells were treated with Tet and VPA for two more days. Knockdown efficiencies of OTULIN were determined by RT-qPCR (c) and by western blotting using an antibody against OTULIN (d), n = 2 biological replicates and 2 technical replicates, mean ± s.d., two-sided t test, * *P* = 0.01567, 0.05357, 0.01388 (siCntrl vs. siOTULIN).

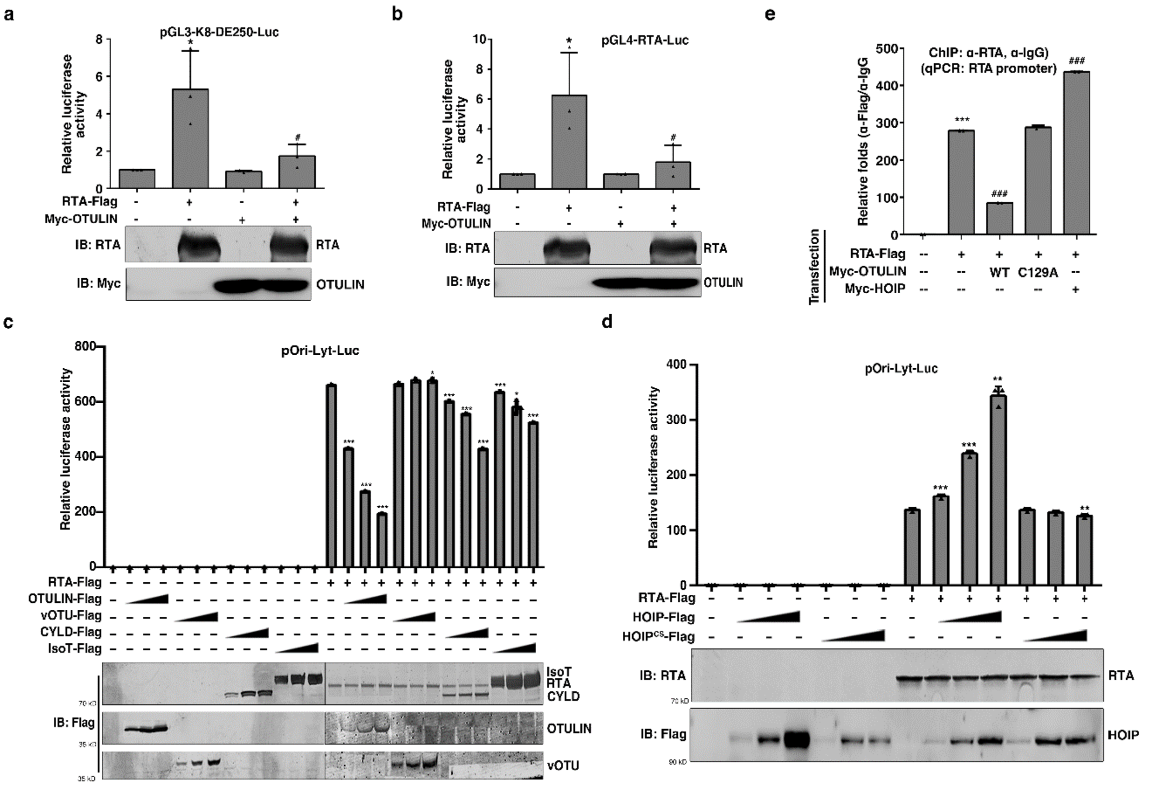

Supplementary Fig. 2. OTULIN and HOIP regulates RTA transcriptional activity (Related to Fig. 2). (**a** and **b**) OTULIN overexpression inhibits RTA transactivation activities. HEK293T cells were transfected with the firefly luciferase reporter constructs pGL3-K8-DE250-Luc (a) or pGL4-RTA-Luc (b), a Renilla luciferase control plasmid, RTA, and OTULIN for reporter assay. a, n = 3 biological replicates and 2 technical replicates, mean ± s.d., two-sided t test, * P = 0.01090 (Cntrl vs. RTA), # P = 0.02215 (RTA vs. RTA+OTULIN); b, n = 3 biological replicates and 2 technical replicates, mean ± s.d., two-sided t test, * P = 0.01667 (Cntrl vs. RTA), # P = 0.03257 (RTA vs. RTA+OTULIN). (**c**) OTULIN inhibits RTA transactivation activity. HEK293T cells were transfected with the firefly luciferase reporter construct pOri-Lyt-Luc, a Renilla luciferase control plasmid, RTA, together with OTULIN or other deubiquitination enzymes (vOTU, CYLD and IsoT)

for reporter assay, n = 3 biological replicates and 2 technical replicates, mean ± s.d., two-sided t test, * P = 0.0000021, 0.0000013, 0.0000028, 0.0256410, 0.0000194, 0.0000026, 0.0000001, 0.0003625, 0.0298672, 0.0000581. (**d**) HOIP enhances RTA transactivation activity. HEK293T cells were transfected with the firefly luciferase reporter construct pOri-Lyt-Luc, a Renilla luciferase control plasmid, RTA, HOIP or HOIP mutant for reporter assay, n = 3 biological replicates and 2 technical replicates, mean ± s.d., two-sided t test, * P = 0.0008165, 0.0000496, 0.0017552, 0.0085083. (**e**) OTULIN inhibits while HOIP enhances RTA binding to the RTA promoter. HEK293T cells were co-transfected with the reporter construct pGL4-RTA-Luc, RTA-Flag, together with Myc-OTULIN, Myc-OTULIN^{C129A} or Myc-HOIP for ChIP assay using anti-Flag magnetic agarose beads. The quantities of RTA promoter DNA in the precipitates were evaluated by qPCR, n = 2 biological replicates and 2 technical replicates, mean ± s.d., two-sided t test, * P < 0.001 (Cntrl vs. RTA), #, P <0.001 (RTA vs. OTULIN+RTA, HOIP, respectively).

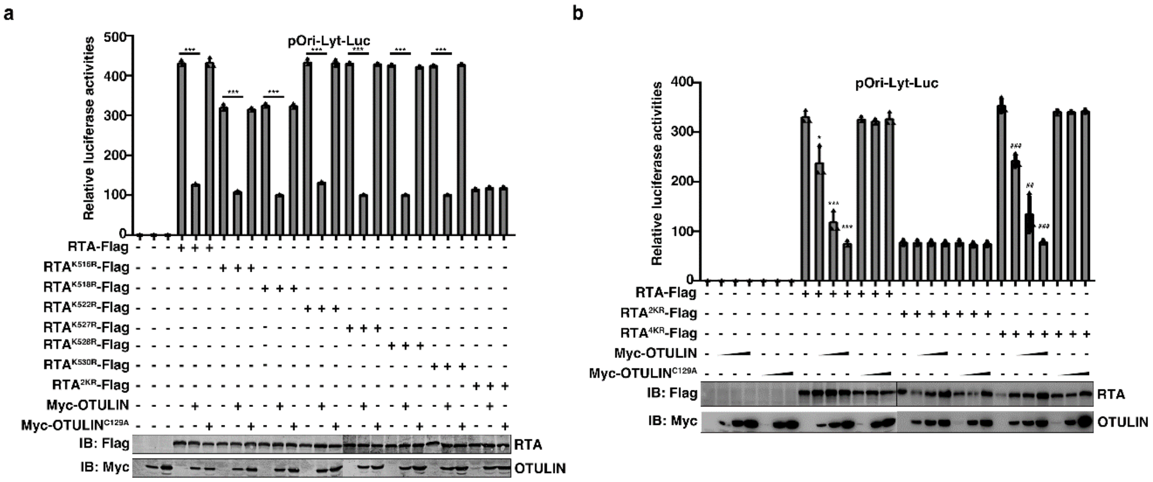

Supplementary Fig. 3. OTULIN inhibits RTA transcriptional activities (Related to Fig. 3). (**a**) RTA^{2KR} is defective in transcriptional activity. RTA and its mutants as shown in Figure 3E were tested for their transcriptional activities in the pOri-Lyt-Luc reporter assays. n = 3 biological replicates and 2 technical replicates, mean ± s.d., two-sided t test, * *P* =0.0000549, 0.0001465, 0.0000296, 0.0001285, 0.0000078, 0.0000057, 0.0000070. (**b**) RTA^{4KR} is not defective in transcriptional activity. WT RTA, RTA^{2KR} and RTA^{4KR} were tested for their transcriptional activities in the pOri-Lyt-Luc reporter assays. n = 3 biological replicates and 2 technical replicates, mean ± s.d., two-sided t test, * *P* = 0.0250056, 0.0004383, 0.0000650 (RTA vs. RTA+OTULIN); # *P* =0.0007671, 0.0041614, 0.0004379 (RTA^{4KR} vs. RTA^{4KR} +OTULIN).

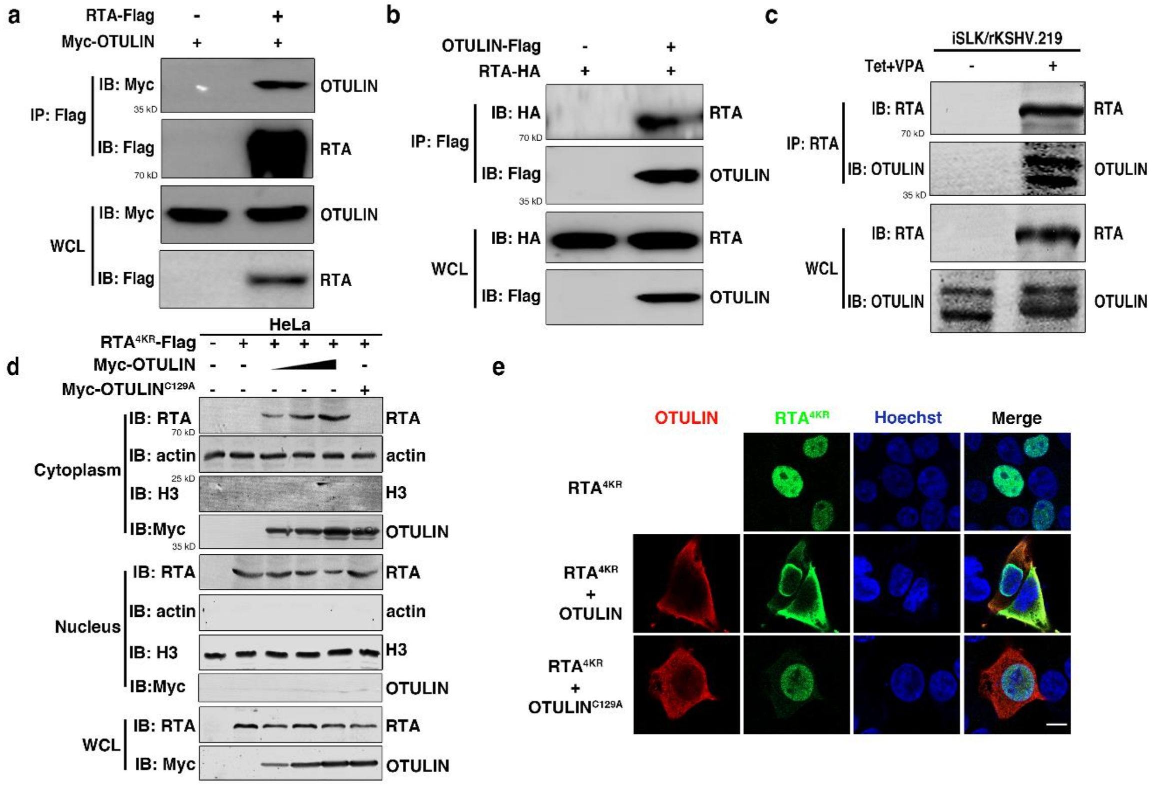

Supplementary Fig. 4. OTULIN interacts with RTA (Related to Fig. 4). (**a** and **b**) OTULIN interacts with RTA. HEK293T cells were co-transfected with the indicated plasmids. After 36 hours cells were harvested and subjected to immunoprecipitation with anti-Flag magnetic agarose beads. Immunoprecipitated proteins were detected with the indicated antibodies. n = 3 independent experiments. (**c**) Endogenous RTA interacts with endogenous OTULIN. iSLK/rKSHV.219 cells were treated with Tet plus VPA for 48 hours. The cells were collected and subjected to immunoprecipitation with anti-RTA antibody. Co-immunoprecipitated OTULIN was detected by western blotting with an anti-OTULIN antibody. n = 3 independent experiments. (**d** and **e**) RTA^{4KR} locates in the nucleus and OTULIN induces its cytoplasmic translocation. HeLa cells were transfected with RTA^{4KR} in the presence or absence of OTULIN or $OTULIN^{C129A}$ and harvested for fractionation into cytoplasmic and nuclear portions and immunoblotting with the indicated antibodies (d). HEK293T cells transfected with the indicated plasmids were subjected to immunofluorescence staining to assess the subcellular localization of RTA^{4KR} (green). Cell nuclei in the fields were stained with Hoechst (blue) (e). Scale bar: 10 µm. Image is representative of n = 3 independent experiments.

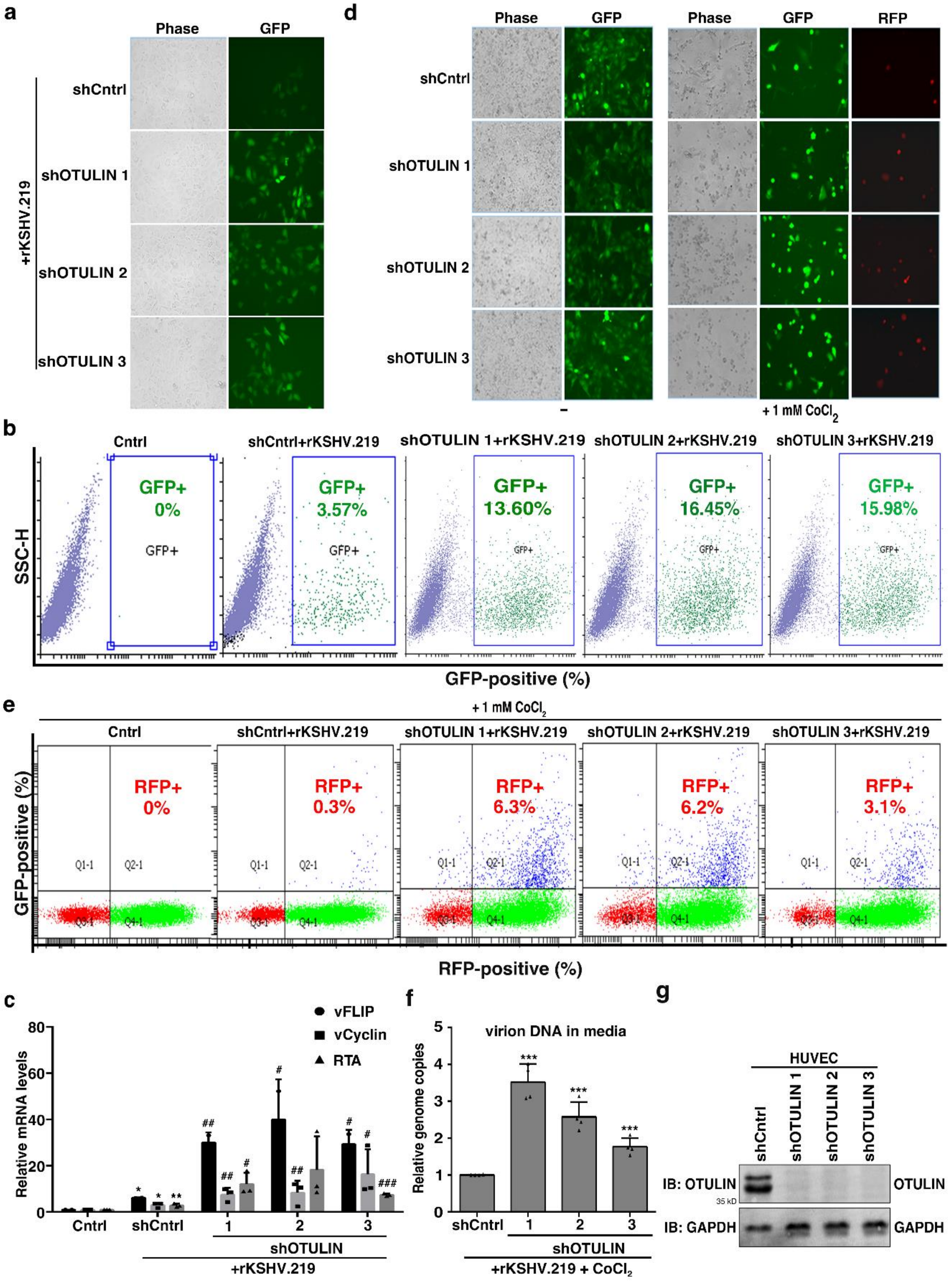

Supplementary Fig. 5. OTULIN inhibits KSHV de novo infection. (**a**) Stable HUVEC

cells with control shRNA (shCntrl) or shRNAs against OTULIN (shOTULIN 1, 2, and 3) were infected with rKSHV.219. Two days later photomicrographs showing phase and GFP fluorescence were taken. Image is representative of n = 3 independent experiments. (**b**) As in (a) but the cells were harvested for flow cytometric evaluation of GFP fluorescence. The gating strategy is shown in Supplementary Fig. 6b. (**c**) As in (a), but cells were collected for RNA extraction and the mRNA levels of KSHV genes *vFLIP*, *vCyclin* and *RTA* were measured by RT-qPCR, *, $P < 0.05$ and **, $P < 0.01$ by two-sided *t*-test versus the Cntrl group, n = 3 biological replicates and 2 technical replicates, mean ± s.d., two-sided t test, * $P = 0.04479$ (shCntrl vs. shCntrl+KSHV), #, $P = 0.00189$, 0.05407, 0.01660 (shCntrl vs. shOTULIN1, 2, 3, respectively) for vFLIP; * $P = 0.03042$ (siCntrl vs. siCntrl+TPA+NaB), #, P =0.03299, 0.02145 (siCntrl vs. siOTULIN1, 3, respectively) for vcyclin; * $P = 0.00567$ (siCntrl vs. siCntrl+TPA+NaB), #, P =0.01808, 0.07076, 0.00049 (siCntrl vs. siOTULIN1, 3, respectively) for RTA. (**d**) Stable OTULIN KD HUVEC cells harboring rKSHV.219 generated from (a) were treated with $CoCl_2$ for two days and photomicrographs showing phase, GFP and RFP fluorescence were taken. Image is representative of n = 3 independent experiments. (**e**) As in (D) but the cells were harvested for flow cytometric evaluation of RFP fluorescence. The gating strategy is shown in Supplementary Fig. 6c. (**f**) As in (d) but the culture media were collected for DNA extraction. The relative quantities of virus particles were determined by qPCR, n = 4 biological replicates and 2 technical replicates, mean ± s.d., two-sided t test, * $P = 0.00002$, 0.00010, 0.00028 (shCntrl vs. shOTULIN). (**g**) Western blot analysis of OTULIN levels in cell lysates of stable OTULIN KD HUVEC

cells.

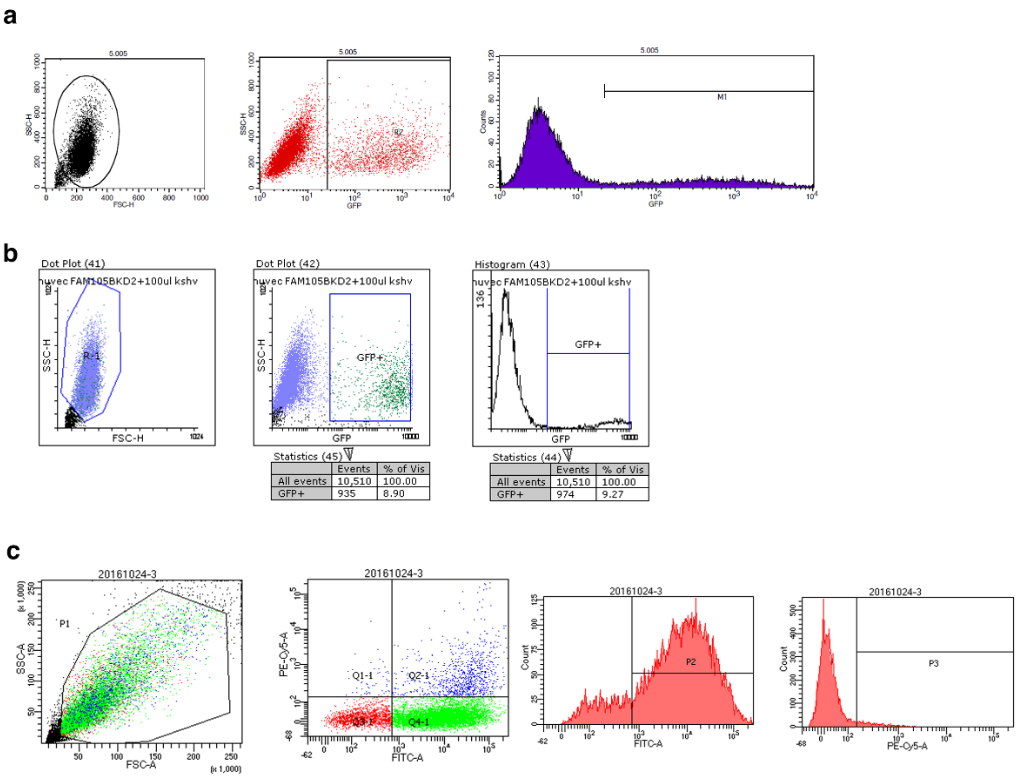

Supplementary Fig. 6. Gating strategy used in flow cytometry analysis. (**a**) Related to Figure1b, e. (**b**) Related to Supplementary Figure 5b. (**c**) Related to Supplementary Figure 5e.
