# Supplementary material for: Linear ubiquitination regulates the KSHV replication and transcription activator protein to control infection
Source: Nat Commun. 2024 Jun 29;15:5515. doi: 10.1038/s41467-024-49887-6 (PMC11217414; doi:10.1038/s41467-024-49887-6)
Supplement: Supplementary file 3 — Reporting Summary [file 41467_2024_49887_MOESM3_ESM.pdf]

nature portfolio

Corresponding author(s): Zongping Xia

Last updated by author(s): May 5, 2024

Reporting Summary

Nature Portfolio wishes to improve the reproducibility of the work that we publish. This form provides structure for consistency and transparency in reporting. For further information on Nature Portfolio policies, see our Editorial Policies and the Editorial Policy Checklist.

Statistics

For all statistical analyses, confirm that the following items are present in the figure legend, table legend, main text, or Methods section.

n/a	Confirmed	
☐	☒	The exact sample size (n) for each experimental group/condition, given as a discrete number and unit of measurement
☐	☒	A statement on whether measurements were taken from distinct samples or whether the same sample was measured repeatedly
☐	☒	The statistical test(s) used AND whether they are one- or two-sided<br>*Only common tests should be described solely by name; describe more complex techniques in the Methods section.*
☒	☐	A description of all covariates tested
☒	☐	A description of any assumptions or corrections, such as tests of normality and adjustment for multiple comparisons
☒	☐	A full description of the statistical parameters including central tendency (e.g. means) or other basic estimates (e.g. regression coefficient) AND variation (e.g. standard deviation) or associated estimates of uncertainty (e.g. confidence intervals)
☒	☐	For null hypothesis testing, the test statistic (e.g. F, t, r) with confidence intervals, effect sizes, degrees of freedom and P value noted<br>*Give P values as exact values whenever suitable.*
☒	☐	For Bayesian analysis, information on the choice of priors and Markov chain Monte Carlo settings
☒	☐	For hierarchical and complex designs, identification of the appropriate level for tests and full reporting of outcomes
☒	☐	Estimates of effect sizes (e.g. Cohen's d, Pearson's r), indicating how they were calculated

Our web collection on statistics for biologists contains articles on many of the points above.

Software and code

Policy information about availability of computer code

Data collection	*Provide a description of all commercial, open source and custom code used to collect the data in this study, specifying the version used OR state that no software was used.*
Data analysis	*Provide a description of all commercial, open source and custom code used to analyse the data in this study, specifying the version used OR state that no software was used.*

For manuscripts utilizing custom algorithms or software that are central to the research but not yet described in published literature, software must be made available to editors and reviewers. We strongly encourage code deposition in a community repository (e.g. GitHub). See the Nature Portfolio guidelines for submitting code & software for further information.

Data

Policy information about availability of data

All manuscripts must include a data availability statement. This statement should provide the following information, where applicable:

- Accession codes, unique identifiers, or web links for publicly available datasets
- A description of any restrictions on data availability
- For clinical datasets or third party data, please ensure that the statement adheres to our policy

The datasets are available within the Article, Supplementary Information, or Source Data file. Source data are provided as a Source Data file. Source data are provided with this paper.

Research involving human participants, their data, or biological material

Policy information about studies with human participants or human data. See also policy information about sex, gender (identity/presentation), and sexual orientation and race, ethnicity and racism.

Reporting on sex and gender	Not applicable
Reporting on race, ethnicity, or other socially relevant groupings	Not applicable
Population characteristics	Not applicable
Recruitment	Not applicable
Ethics oversight	Not applicable

Note that full information on the approval of the study protocol must also be provided in the manuscript.

Field-specific reporting

Please select the one below that is the best fit for your research. If you are not sure, read the appropriate sections before making your selection.

☒ Life sciences ☐ Behavioural & social sciences ☐ Ecological, evolutionary & environmental sciences

For a reference copy of the document with all sections, see nature.com/documents/nr-reporting-summary-flat.pdf

Life sciences study design

All studies must disclose on these points even when the disclosure is negative.

Sample size	No statistical methods were used to determine sample size. The number of biological replicates are indicated in the figure legends.
Data exclusions	No data were excluded from the analysis.
Replication	All replication attempts were successful.
Randomization	No randomization done for these experiments.
Blinding	Different groups were evaluated blindly.

Reporting for specific materials, systems and methods

We require information from authors about some types of materials, experimental systems and methods used in many studies. Here, indicate whether each material, system or method listed is relevant to your study. If you are not sure if a list item applies to your research, read the appropriate section before selecting a response.

Materials & experimental systems

n/a	Involved in the study	
☐	☒	Antibodies
☐	☒	Eukaryotic cell lines
☒	☐	Palaeontology and archaeology
☒	☐	Animals and other organisms
☒	☐	Clinical data
☒	☐	Dual use research of concern
☒	☐	Plants

Methods

n/a	Involved in the study	
☒	☐	ChIP-seq
☐	☒	Flow cytometry
☒	☐	MRI-based neuroimaging

Antibodies

Antibodies used	RTA (8C12, Argene, 1:1000), Myc (Huabio, 0912-2, Rabbit, 1:1000), Flag (Huabio, 0912-1, Rabbit, 1:1000), Linear Ubiquitin (Millipore, MABS199, Rabbit, 1:1000), Histone H3 (Huabio, HA500298, Rabbit, 1:1000), β-actin (Huabio, R1207-1, Rabbit, 1:1000), OTULIN (Abcam, ab151117, 1:1000) and GAPDH (Cell Signaling Technology, 2118, Rabbit, 1:10000)

Validation	RTA (8C12, Argene) is validated on manufacturer website for western blot. We also validated this antibody in cells expressing FLAG-RTA protein.<br>OTULIN (Abcam, ab151117, 1:1000), Myc (Huabio, 0912-2, AB_3068716), Flag (Huabio, 0912-1, AB_3068715), Histone H3 (Huabio, HA500298, AB_3071393), β-actin (Huabio, R1207-1, AB_3073201) are validated on manufacturer website for western blot.<br>Linear Ubiquitin (Millipore, MABS199) validated on manufacturer website for western blot.<br>GAPDH (Cell Signaling Technology, 2118) validated on manufacturer website for western blot.

Eukaryotic cell lines

Policy information about cell lines and Sex and Gender in Research

Cell line source(s)	iSLK/rKSHV.219 cells were provided by Dr. Dr. Ke Lan (Wuhan University), BCBL-1 cells were provided by Dr. Qiming Liang (Shanghai Jiao Tong University), HEK293T cells (CRL-3216), HeLa cells (CCL-2), and human umbilical vein endothelial cells (HUVECs, CRL-1730) were obtained from ATCC.
Authentication	Cell lines were obtained from investigators who generated them, and then passed through an intermediary, or generated in our lab. They are not further authenticated.
Mycoplasma contamination	All cell lines used in this study were mycoplasma free. Cell lines were routinely tested for mycoplasma by PCR.
Commonly misidentified lines<br>(See ICLAC register)	None

Plants

Seed stocks	Not applicable
Novel plant genotypes	Not applicable
Authentication	Not applicable

Flow Cytometry

Plots

Confirm that:

☒ The axis labels state the marker and fluorochrome used (e.g. CD4-FITC).

☒ The axis scales are clearly visible. Include numbers along axes only for bottom left plot of group (a 'group' is an analysis of identical markers).

☒ All plots are contour plots with outliers or pseudocolor plots.

☒ A numerical value for number of cells or percentage (with statistics) is provided.

Methodology

Sample preparation	iSLK/rKSHV.219 cells were treated with 1 μg/ml tetracycline plus 1 mM valproate (VPA) (Sigma) for 48hours to activate lytic replication. To evaluate RFP+ or GFP+ in cells by Flow cytometry, cells were washed and resuspended with PBS. Events were acquired using FACSCalibur (BD), and data were analyzed using FlowJo software. Gating was performed by comparison with control cells.
Instrument	iSLK/rKSHV.219 cell flow cytometry analysis was performed on a FACSCalibur (BD).
Software	FlowJo software
Cell population abundance	Not applicable
Gating strategy	We gated on GFP positive cells and then gated on RFP positive cells.

☒ Tick this box to confirm that a figure exemplifying the gating strategy is provided in the Supplementary Information.
